# Precise Segmentation of COVID-19 Infected Lung from CT Images Based on Adaptive First-Order Appearance Model with Morphological/Anatomical Constraints

**DOI:** 10.3390/s21165482

**Published:** 2021-08-14

**Authors:** Ahmed Sharafeldeen, Mohamed Elsharkawy, Norah Saleh Alghamdi, Ahmed Soliman, Ayman El-Baz

**Affiliations:** 1 BioImaging Laboratory, Department of Bioengineering, University of Louisville, Louisville, KY 40292, USA; a.sharafeldeen@louisville.edu (A.S.); mohamed.elsharkawy@louisville.edu (M.E.); ahmed.soliman@louisville.edu (A.S.); 2 College of Computer and Information Science, Princess Nourah Bint Abdulrahman University, Riyadh 11564, Saudi Arabia

**Keywords:** computed tomography (CT), lung, chest, segmentation, COVID-19

## Abstract

A new segmentation technique is introduced for delineating the lung region in 3D computed tomography (CT) images. To accurately model the distribution of Hounsfield scale values within both chest and lung regions, a new probabilistic model is developed that depends on a linear combination of Gaussian (LCG). Moreover, we modified the conventional expectation-maximization (EM) algorithm to be run in a sequential way to estimate both the dominant Gaussian components (one for the lung region and one for the chest region) and the subdominant Gaussian components, which are used to refine the final estimated joint density. To estimate the marginal density from the mixed density, a modified k-means clustering approach is employed to classify the Gaussian subdominant components to determine which components belong properly to a lung and which components belong to a chest. The initial segmentation, based on the LCG-model, is then refined by the imposition of 3D morphological constraints based on a 3D Markov–Gibbs random field (MGRF) with analytically estimated potentials. The proposed approach was tested on CT data from 32 coronavirus disease 2019 (COVID-19) patients. Segmentation quality was quantitatively evaluated using four metrics: *Dice similarity coefficient (DSC)*, *overlap coefficient*, *95th-percentile bidirectional Hausdorff distance (BHD)*, and *absolute lung volume difference (ALVD)*, and it achieved $95.67_{\pm 1.83}\%$, $91.76_{\pm 3.29}\%$, $4.86_{\pm 5.01}$, and $2.93_{\pm 2.39}$, respectively. The reported results showed the capability of the proposed approach to accurately segment healthy lung tissues in addition to pathological lung tissues caused by COVID-19, outperforming four current, state-of-the-art deep learning-based lung segmentation approaches.

## 1. Introduction

Pulmonary diseases are serious public heath threats that may happen after having inflammation or fluid accumulation in the lung, causing a respiratory failure, such as coronavirus disease 2019 (COVID-19). The primary reason for COVID-19 death is acute respiratory distress syndrome (ARDS) [1]. According to Gupta et al. [2], 83.9% of the COVID-19 patients in their study needed a mechanical ventilation support, of whom 87.95% had ARDS. Therefore, detection and diagnosis of COVID-19 grades is vital to prioritize patient’s need for ventilator support. The accuracy attainable by computer-aided diagnostic (CAD) system using lung imaging data for COVID-19 depends on how accurate the segmentation is. Accurate lung segmentation is a challenging task as different pathologies affect the appearance of the lung, and if the infected regions are missed during the segmentation, it will affect the entire task. Therefore, this paper focuses on developing an automatic system to detect and segment the lungs in chest computed tomography (CT), which is one of the popular noninvasive clinical modalities used by physicians to diagnose lung pathologies.

In the last few years, many preliminary studies have been conducted to detect and segment lung as well as pathological lesions. Some of these studies [3,4,5,6,7,8,9] proposed threshold-based approaches for lung segmentation, which performed well on normal CT scans but failed in pathological cases, especially severe cases, whereas lungs in the normal CT scan can be discriminated easily from background due to huge differences in attenuation [10]. Therefore, to overcome this problem, more recent studies employed texture, shapes, deep learning, or hybrid techniques to accurately segment normal and different lung pathologies. These studies are briefly discussed below.

In [11,12,13,14], authors considered texture analysis, shape analysis, or both of them in their system to discriminate between objects. A recent study by Oulefki et al. [15] proposed a system to automatically segment COVID-19 lung infected region by applying a multilevel entropy-based threshold approach, namely a modified Kapur method. Their system achieved a sensitivity, specificity, Dice similarity coefficient (DSC), and precision of 73.3%, 99.4%, 71.4%, and 73.9%, respectively. Another study by Korfiatis et al. [16] employed k-means clustering to partition CT voxels into four classes: lung, muscle, fat, and bone based on intensity values. After that, the initial lung region was extracted by applying a filling operation. Finally, a support vector machine (SVM) was used to determine the final border of the lung based on intensity and wavelet-based descriptors. In [17], authors proposed a segmentation system by eliminating unwanted regions and segmenting lung initially using a threshold approach. Moreover, a 3D gray-level co-occurrence matrix (GLCM) was constructed for a window of size 15×15×15 centered on each voxel. Then, predefined features were extracted from the GLCM, and a new image was constructed, being the product of the entropy and the inverse difference moment of the GLCM. Subsequently, the abnormal regions were identified from the constructed image using a threshold approach. Finally, the later and initial segmentation were merged together to determine the final segmentation. Dehmeshki et al. [18] used a genetic algorithm (GA) to construct a system to identify spherical nodules within CT images. First, the lung was segmented using adaptive thresholding. Then, the authors utilized a geometric feature, namely, volumetric shape index (VSI), for the segmented lung as a weighted factor in the fitness function of GA. VSI of a spherical object is 1, while that of a cylindrical object is 0.75, so the values of fitness function for nodules were higher than for blood vessels. Convergence criteria of GA to select the shape as a nodule was a threshold-based. The detection rate of their system was approximately 90% with a 14.6 false positive per scan. Moreover, Nakagomi [19] presented a min-cut graph segmentation algorithm based on multiple shapes and prior information of neighbors structure to detect and segment lung infected by pleural effusion. In [20], authors presented a lung segmentation system for different lung pathologies. Their system first determined two seed points within both lungs using a thresholding approach, then a fuzzy connectedness (FC) algorithm was used to extract the lung. Furthermore, multiple refinement stages based on machine learning classification and neighboring anatomy-guided learning mechanisms were included in their system to detect pathological regions during FC segmentation. A recent study by Houssein et al. [21] developed a segmentation system that employed a heuristic method, called manta ray foraging optimization (MRFO), based on an opposition-based learning (OBL), using Otsu’s method as a fitness function, to get the best threshold values using COVID-19 CT images. More information about texture- and shape-based lung segmentation can be found in [22].

Recently, deep learning approaches have been employed to segment normal as well as pathological lung caused by COVID-19. For example, Saood et al. [23] investigated two deep learning approaches to semantically segment infected/non-infected lung using CT images. These included SegNet [24] and U-Net [25] networks. The author employed these networks for binary and multi-class classification. They conducted multiple experiments with different hyperparameters. The best reported results for the binary (multi-class) classification gave accuracy of $95.4_{\pm 2.9}\%$ ($90.7_{\pm 6}\%$) and $94.9_{\pm 4.3}\%$ ($90.8_{\pm 6.5}\%$) using SegNet and U-Net, respectively. A similar study [26] proposed a segmentation system using a convolution neural network (CNN). Their network employed feature variation block to enhance the efficiency of feature representation as well as progressive atrous spatial pyramid pooling to deal with appearance and shape differences caused by sophisticated infection. The DSC (sensitivity, specificity) of their system was 72.6% (75.1%, 72.6%) and 98.7% (98.6%, 99%) for COVID-19 infections and normal lung CT images, respectively. A multi-task deep learning-based system was implemented by Amyar et al. [27]. This study included reconstruction for better feature representation; segmentation to extract lesion regions; and classification to categorize the scan into normal, COVID-19, and other diseases. Their system employed encoder-decoder architecture based on U-Net network which used a common encoder for the three tasks. The best reported DSC of their segmentation task was 88%. Recent study by Fan et al. [28] developed a binary and multi-class segmentation system using CT chest images, called Inf-Net. This system was mainly based on a deep learning. Moreover, to compensate the limited number of labeled images, they included a random sampling-based semi-supervised learning, namely, Semi-Inf-Net. Their system employed edge attention as well as reverse attention to improve the feature representation by modeling lung boundaries. In addition, high-level features were exploited by their network and combined by a parallel partial decoder. The performance of their infection segmentation system achieved a DSC of 68.2% and 73.9%, sensitivity of 69.2% and 72.5%, and specificity of 94.3% and 96% using Inf-Net and Semi-Inf-Net, respectively. A similar study [29] proposed an automatic deep learning-based multi-class segmentation system of COVID-19 using CT chest images. The latter exploited aggregated residual transformations in addition to soft attention mechanism to better represent the features and to increase the system’s ability to distinguish between different COVID-19 lesions. The reported DSC (accuracy, precision) of their system was 94% (89%, 95%) and 83% (79%, 82%) with and without data augmentation, respectively. Another study [30] proposed a semi-supervised deep learning-based segmentation system, called FSS-2019-nCov, to detect lesion infection in COVID-19 patients. The latter was based on encoder–decoder architecture with Res2Net [31] encoder backbone. In the proposed encoder–decoder architecture, the authors used a context enrichment module, namely, smoothed atrous convolution block and the multi-scale pyramid pooling block, to overcome any debilitation occurred in the represented knowledge generated in the encoder phase. This system was consisted of three modules: conditioner path, adaptive interaction module, and segmentation path. Conditioner path was responsible to learn feature maps from support sets which contain CT slice and its ground-truth. Subsequently, these feature maps were transmitted to the segmentation path using adaptive interaction module which was responsible for detecting lesion in the CT slice. The performance of their system achieved a DSC of 79.8%, sensitivity of 80.3%, and specificity of 98.6%. In [32], the authors employed V-Net [33] to segment lung in COVID-19 CT images that was refined by a shape deformation module. A similar study by Li et al. [34] employed U-Net to segment lung on CT images. Then, they proposed a deep learning network, called COVNet, with a ResNet-50 [35] backbone to detect COVID-19 lesions. A recent study [36] developed a deep learning-based segmentation system, called LungINFseg, to detect COVID-19 lesions in CT images. This system was built on the basis of encoder–decoder architecture. The authors employed a 2D discrete wavelet transform (DWT) with four Haar filters and a receptive field aware (RFA) module in the encoder phase, which were able to change the size of receptive field, to capture more relevant features related to infected regions. Their system achieved a DSC and intersection over union (IoU) score of 80.34% and 68.77%, respectively. Other studies have also employed deep learning as a segmentation system with varying accuracy as reported in [36,37,38,39,40,41,42,43].

Segmentation techniques for CT data using deep learning method consider the current, state-of-the-art approaches. However, they have some drawbacks in practical applications, such as the need for huge databases to learn the different pathology of the lung regions which makes the training of such network is very high computational [44]. Moreover, segmentation approaches based on deformable models, which optimize a trade-off between smoothness of the deformable boundary and homogeneity of the region inside the boundary, suffer from high computational complexity and limited capabilities when the desired boundary has concavities or encompasses a region that is naturally inhomogeneous, such as infected lung regions. To overcome the aforementioned limitations, we are proposing an unsupervised lung segmentation approach that is based on modeling the first-order appearance model of CT data by using a probabilistic model based on a linear combination of Gaussian (LCG) that estimates dominant components, corresponding to lung and chest regions, as well as subdominant components. Subsequently, these subdominant components are clustered to one of the dominant components for marginal density estimation. This model can capture the variability in the Hounsfield distributions that may come from changing the screening protocols and severity of lung infections. Finally, we refine the lung segmentation by applying 3D morphological constraints based on the Markov–Gibbs random field (MGRF) model with analytical parameter estimations.

## 2. Methods

**Figure 1:**
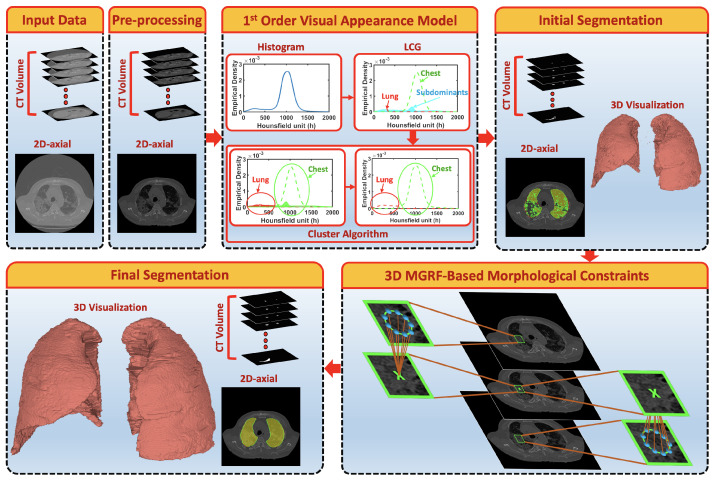
Schematic illustration of the pipeline of the proposed segmentation system using CT images.

A fully automated segmentation framework is presented to extract both healthy lung tissues as well as pathological lung tissues that may be caused by COVID-19. The major steps of the framework, depicted in Figure 1, are as follows: (i) preprocessing 3D chest CT scans to identify background voxels; (ii) modeling the gray-level distribution of the CT data as a Gaussian mixture model with parameters estimated using a novel, sequential, expectation-maximization (EM)-based approach; (iii) preliminary segmentation of the lung region based on the use of a Bayes classifier; and (iv) refining the segmentation using a three-dimensional, rotation- and translation-invariant MGRF to impose morphological constraint. Below, we will describe the details of each step.

### 2.1. First-Order Visual Appearance Model

**Figure 2:**
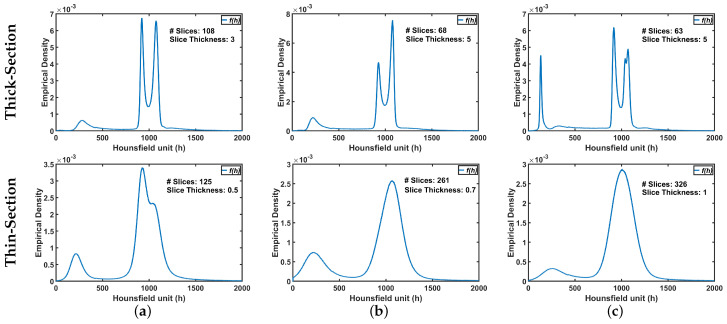
An illustrative example of variability of CT appearance (distribution of radiodensities) for (**a**) healthy/mild, (**b**) moderate, and (**c**) severe COVID-19 infections.

The ultimate goal is accurate labeling of voxels as belonging to lung tissue or background, where accuracy is defined as close agreement with “ground-truth” lung region delineated by a radiologist. The main challenge in modeling the distribution of the radiodensities (in Hounsfield units) of lung and chest tissues, i.e., the relative frequency histogram of CT voxel values, is dependent upon slice thickness and the severity of lung infection as shown in Figure 2.

To address this challenge, we will assume that the first-order visual appearance model of the CT data (H) can be modeled with linear combination of Gaussian distributions with K≥2 components [45]. The first two components, called the dominant modes, corresponding to the lung region (k=1) and the chest region exterior to the lungs (k=2). The remaining Gaussian components k=3,…,K are called subdominant modes. Thus, the proposed probabilistic model is

(1) $$p\left(h\right)=w_{1}\varphi \left(h;\theta_{1}\right)+w_{2}\varphi \left(h;\theta_{2}\right)+\sum_{k=3}^{K}w_{k}\tau \left(h;\theta_{k}\right),$$

where the $w_{k}>0$ are mixing weights, and φ is a Gaussian density with parameters $\theta_{k}=\left(\mu_{k},\sigma_{k}\right)$. In order for p(h) to be a density function, the weights must satisfy the constraint

(2) $$\sum_{k=1}^{K}w_{k}=1.$$

Given the number *K* of Gaussian components, the 3K parameters of Equation (1), including mixing weights W and means and variances Θ, are estimated by maximizing the log-likelihood of the empirical data

(3) $$L\left(W,\Theta \right)=\sum_{h=0}^{H}n\left(h\right)\log p\left(h;W,\Theta \right),$$

where n(h) is the histogram of the CT data, whose voxel values range from 0 to *H*. The corresponding relative frequency histogram is denoted f(h)=n(h)/N, *N* being the total number of voxels. To maximize the likelihood in Equation (3), we employ an iterative block relaxation process as follows.

Let τ indicate an iteration such that $\left(W^{\left[\tau \right]},\Theta^{\left[\tau \right]}\right)$ are the parameter estimates on that iteration, and 

(4) $$p^{\left[\tau \right]}\left(h\right)=p\left(h;W^{\left[\tau \right]},\Theta^{\left[\tau \right]}\right)=\sum_{k=1}^{K}w_{k}^{\left[\tau \right]}\tau \left(h;\theta_{k}^{\left[\tau \right]}\right)$$

is the proposed probabilistic model for the CT data. The conditional weights are estimated as follows:

(5) $$\pi^{\left[\tau \right]}\left(k|h\right)=\frac{w_{k}^{\left[\tau \right]}\tau \left(h;\theta_{k}^{\left[\tau \right]}\right)}{p^{\left[\tau \right]}\left(h\right)};$$

This conditional probability specifies the relative contributions of voxel value *h* to each component at step τ. Using these variables, Equation (3) can be written in the equivalent form:

(6) $$L\left(W^{\left[\tau \right]},\Theta^{\left[\tau \right]}\right)=\sum_{h=0}^{H}n\left(h\right)\log\left[\sum_{k=1}^{K}\pi^{\left[\tau \right]}\left(k|h\right)\tau \left(h;\theta_{k}^{\left[\tau \right]}\right)\right]$$

From given starting values at τ=0, the block relaxation scheme converges to a local maximum of the likelihood function in Equation (6) through iteration of the following two steps:

1. E-step [τ+1]: estimate $W^{\left[\tau +1\right]}$, $\Theta^{\left[\tau +1\right]}$, which maximize L(W,Θ) under the fixed conditional weights of Equation (5) at step τ.
2. M-step [τ+1]: recalculate weights, which maximize *L* holding parameters $W^{\left[\tau +1\right]}$ and $\Theta^{\left[\tau +1\right]}$ fixed.

The process is repeated until the changes of all the parameters become small.

The E-step maximizes the likelihood function of Equation (6) subject to the constraints Equation (2). The solution for the weights is

(7) $$w_{k}^{\left[\tau +1\right]}=\sum_{h=0}^{H}f\left(h\right)\pi^{\left[\tau \right]}\left(k|h\right)$$

Then, parameters of each Gaussian component are found using the ordinary (unconstrained) maximum likelihood estimates:

(8) $$\begin{array}{c} \mu_{k}^{\left[\tau +1\right]}=\frac{1}{w_{k}^{\left[\tau +1\right]}}\sum_{h=0}^{H}h\cdot f\left(h\right)\pi^{\left[\tau \right]}\left(k|h\right)\\ {\left(\sigma_{k}^{\left[\tau +1\right]}\right)}^{2}=\frac{1}{w_{k}^{\left[\tau +1\right]}}\sum_{h=0}^{H}{\left(h-\mu_{k}^{\left[\tau +1\right]}\right)}^{2}\cdot f\left(h\right)\pi^{\left[\tau \right]}\left(k|h\right) \end{array}$$

We will follow Algorithm 1 to illustrate the steps for estimating the parameters of the proposed probabilistic model. The final estimated density will consist of the two dominant Gaussian components and K−2 subdominant Gaussian components. Jensen–Shannon divergence (JSD) [46] is employed in this algorithm to measure the similarity between empirical density and mixed density for use as convergence criteria to determine the number of Gaussian components. The latter is a symmetric version of a Kullback–Leibler divergence [47].
**Algorithm 1:** Estimation of the proposed probabilistic model parameters
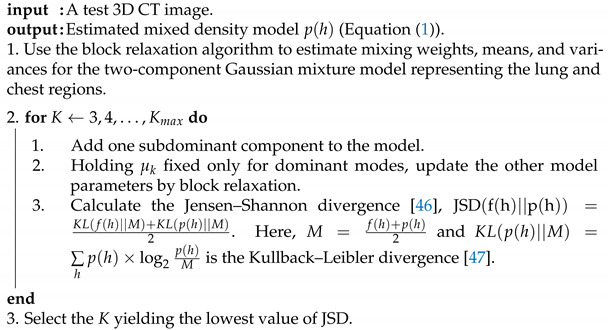


**Estimation of the marginal density:** The K−2 subdominant components of the final estimated model p(h) need to be partitioned among the two dominant modes. Each subordinate component is associated with one dominant component in order to minimize the expected misclassification rate. This is accomplished using the proposed Algorithm 2.
**Algorithm 2:** The proposed clustering algorithm
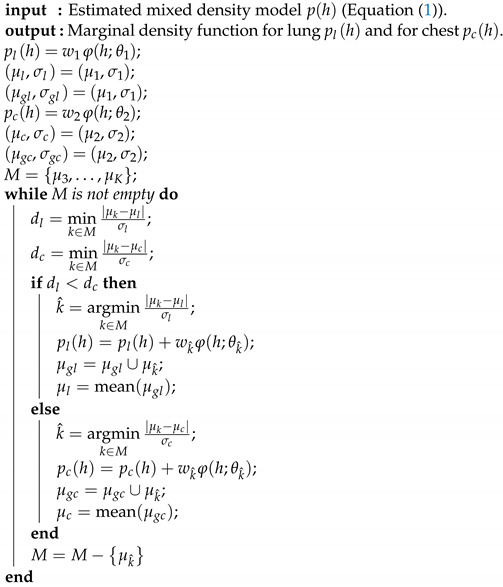


### 2.2. MGRF-Based Morphological Constraints

**Figure 3:**
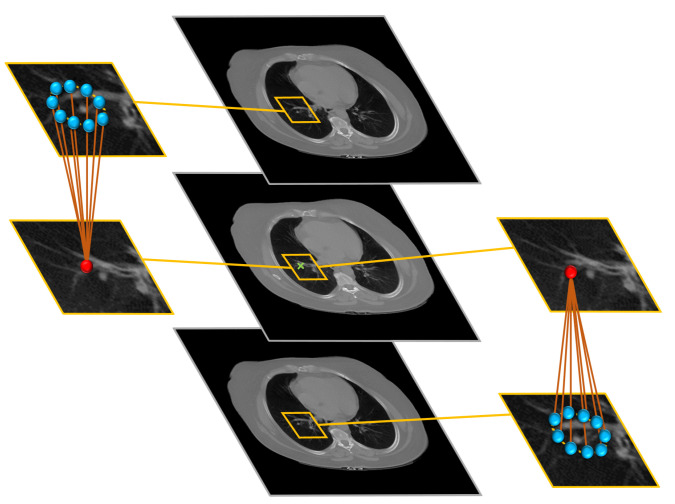
Illustration of the 3D MGRF-Based morphological constraints on the anatomical segmentation. The middle column shows the selected slice, and its upper and lower slices; the left column shows the selected pixel and its neighbors at the upper slice while the right column shows the selected pixel and its neighbors at the lower slice.

To get a consistent segmentation of the lung region, we applied rotation invariant spatial constraints by using a generic Markov–Gibbs model of region maps [45]. The model, which incorporates voxel–voxel interaction effects as shown in Figure 3, has, in general, an arbitrary interaction structure and corresponding Gibbs potentials. For simplicity, we restrict the interaction neighborhood system (N) to the nearest 9 neighbors in the above CT slice and 9 neighbors in the below CT-slice.

To model the interactions between the CT voxels, we will assume all the interactions as the same within each region. To estimate this interaction in analytical way, let $V:X\times X\to \{V_{\mathrm{eq}},V_{\mathrm{ne}}\}$ denote a bi-valued Gibbs potential describing pairwise interactions, where

(9) $$V\left(x,\chi \right)=\left\{\begin{array}{cc} V_{\mathrm{eq}} & x=\chi \\ V_{\mathrm{ne}} & x\neq \chi \end{array}\right.$$

Then, the Gibbs probability distribution (GPD) of region maps on the 3D lattice R is as follows [45]:

(10) $$P\left(m\right)\propto \exp\left(\sum_{(i,j,z)\in R}\sum_{(\xi ,\eta ,\zeta )\in N}V\left(m_{i,j,z},m_{i+\xi ,j+\eta ,z+\zeta }\right)\right)$$

By modifying the derivation scheme in [45] to fit our model, the following first approximation of the maximum likelihood estimator (MLE) of the potential values for a given map m is obtained:

(11) $$\begin{array}{c} \begin{array}{c} V_{\mathrm{eq}}=\frac{X^{2}}{X-1}\left(f\prime \left(m\right)-\frac{1}{X}\right)\\ V_{\mathrm{ne}}=\frac{X^{2}}{X-1}\left(f\prime \prime \left(m\right)-1+\frac{1}{X}\right) \end{array} \end{array}$$

where f′(m) and f″(m) denote the relative frequency of the equal and non-equal pairs of the labels in all the equivalent voxels pairs $\left\{((i,j,z),(i+\xi ,j+\eta ,z+\zeta )):\;(i,j,z)\in R\right.$; (i+ξ,j+η,z+ζ)∈R; $\left.(\xi ,\eta ,\zeta )\in N\right\}$, respectively.

### 2.3. Joint MGRF Model and Lung Segmentation Algorithm

In order to integrate the first-order appearance model with the spatial probabilistic model that describes the morphological/anatomical constrains, we will assume that the CT data (g) consisting of visual appearance model and its spatial map (m, data labels) follow the following two-level MGRF model:

(12) P(g,m)=P(m)P(g|m)

Here, P(m) is an unconditional distribution of maps that is modeled by MGRF probabilistic model that is demonstrated in Equation (10). P(g|m) is a conditional distribution of gray levels for a given labeling. The Bayesian maximum a posteriori estimation (MAP) of the labeling, given the image g, $m^{*}=\underset{m}{\mathrm{argmax}}L\left(g,m\right)$ maximizes the log-likelihood,

(13) L(g,m)=logP(g|m)+logP(m).

In order to summarize the proposed segmentation system, the basic steps are demonstrated in Algorithm 3.
**Algorithm 3: **Lung Extraction Algorithm**input**: A test 3D CT image.
    **output**: Final 3D lung segmentation.
**1st Order Density Estimation:** Estimate the marginal density function for lung ($p_{l}\left(h\right)$) and marginal density function for chest ($p_{c}\left(h\right)$).**Initial Segmentation/Labeling:** Use Bayes classifier to delineate the initial lung region by using the marginal estimated densities.**Estimation of Gibbs Potentials:** Applying Equation (11) on the initial segmentation to estimate the Gibbs potentials.**Refine Segmentation:** Use iterative conditional mode (ICM) algorithm [45] to find the map that maximize the likelihood of joint MGRF model shown in Equation (13).

## 3. Evaluation Metrics

This section describes the metrics used to gauge the performance of our proposed system: *Dice similarity coefficient (DSC)*, *overlap coefficient*, and *absolute lung volume difference (ALVD)*. Each of these quantifies in some way either the agreement or dissimilarity between the segmentation algorithm result and the corresponding ground-truth segmentation. More detailed explanation is presented in Section 3.1, Section 3.2 and Section 3.3, respectively. Furthermore, a fourth metric, the *95th-percentile bidirectional Hausdorff distance (BHD)* (Section 3.4), is employed to quantify the accuracy of the boundary of the segmented region relative to ground-truth.

### 3.1. Dice Similarity Coefficient (DSC)

**Figure 4:**
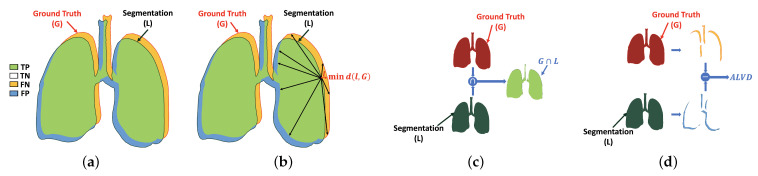
Illustration of the evaluation metrics: (**a**) DSC, (**b**) HD, (**c**) overlap coefficient, and (**d**) ALVD. Note that TP, TN, FP, and FN are true positive (correct lung pixel), true negative (correct background pixel), false positive (incorrect lung pixel), and false negative (incorrect background pixel).

Dice similarity coefficient (DSC) is one of the most common similarity metric to measure the similarity between two different areas. This metric is used to evaluate the result of the proposed system by estimating the similarity between the black-white segmented lung (*L*) and the ground-truth (*G*), i.e., the percentage of common region (i.e., the green part) in both images as shown in Figure 4a. The range of this metric is between 0 and 1, as 0 and 1 mean dissimilar and similar, respectively. It is computed as follows:

(14) $$DSC=\frac{2\times n\left(L⋂G\right)}{n\left(L\right)+n\left(G\right)}$$

where $n\left(L⋂G\right)$ is the cardinality of white pixels in the intersection between the segmented lung (*L*) and the ground-truth (*G*), while $n\left(L\right)$ and $n\left(G\right)$ are the cardinality of the white pixels in the segmentation (*L*) and the ground-truth (*G*), respectively.

### 3.2. Overlap Coefficient

Overlap coefficient is used in our assessment pipeline to measure the similarity between the predicted object and its ground-truth by computing the overlap percentage between them, see Figure 4c. The overlap coefficient of identical objects gives 1, while it gives 0 for heterogeneous one. The latter is estimated as follows:

(15) $$overlap=\frac{n\left(L⋂G\right)}{n\left(L⋃G\right)}$$

where $n\left(L⋃G\right)$ is the cardinality of white pixels in the union between the segmented lung (*L*) and the ground-truth (*G*).

### 3.3. Absolute Lung Volume Difference (ALVD)

Another metric used to assess our work is absolute lung volume difference (ALVD). ALVD computes the similarity between two images by measuring the differences between the ground-truth (*G*) and the black-white segmented lung (*L*) (Figure 4d). The ALVD of similar objects gives 0. This metric is defined as

(16) $$ALVD=\frac{\left|n\left(G\right)-n\left(L\right)\right|}{n\left(G\right)}$$

where $\left|n\left(G\right)-n\left(L\right)\right|$ is the absolute difference between the cardinality of white pixels in the ground-truth (*G*) and segmentation (*L*).

### 3.4. Bidirectional Hausdorff Distance (BHD)

This section describes the last metric called bidirectional Hausdorff distance (BHD), which is used to evaluate our proposed system in addition to the previous three metrics. BHD is the bidirectional estimation of Hausdorff distance (HD) between the black-white segmented lung (*L*) and the ground-truth (*G*), and vice versa. HD is the maximum Euclidean distance between the points in the border of the black-white segmented lung (*L*) and its closest point in the border of the ground-truth (*G*), as visualized in Figure 4b, which is computed as follows [48,49]:

(17) $$HD\left(L,G\right)=\max_{l\in L}\left\{\min_{g\in G}\left\{d\left(l,g\right)\right\}\right\}$$

where *l* and *g* are sets of the points border in the *L* and *G*, respectively, and d(g,l) is the Euclidean distance between the two points.

As, BHD(L,G) is estimated as

(18) BHD(L,G)=max{HD(L,G),HD(G,L)}

In this paper, the 95th-percentile BHD is used to evaluate our proposed system. Instead of getting the maximum Euclidean distance between *L* and *G*, 95th-percentile of all computed distances is selected to overcome the outliers.

## 4. Experimental Results

The segmentation framework described above was applied to the problem of segmenting lung with pathological tissue in COVID-19 patients. The proposed segmentation system is evaluated and tested on 32 CT chest volume with different severity of COVID-19 infections, selected from 249 CT volume in COVID-19 [50]. Four of them had healthy/mild COVID-19 infections whose image size ranges from 512×512×51 to 512×512×125, while 17 patients of size 512×512×36−607 who had moderate infections as well as 11 CT chest volume of size 512×512×44–577 had severe COVID-19 infections. To compare our framework with other approaches that depend on a training dataset, we select another 34 3D CT chest volume (i.e., 3713 images in total) from the same dataset to use them as a training. Table 1 summarized the dataset characteristics used in our experimental results. The data are graded according to the radiology protocol in [51]. To obtain more accurate segmentation, we included morphological/anatomical constraints based on the use of a rotation invariant MGRF model.

**Figure 5:**
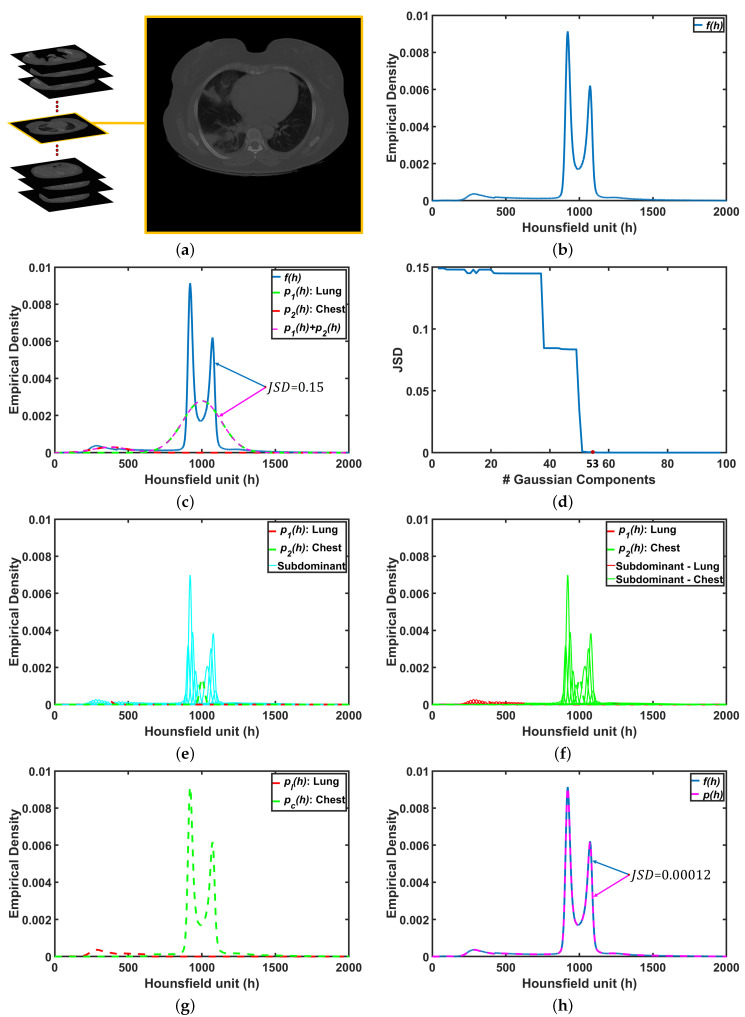
An illustrative example of the proposed system: (**a**) CT chest volume, (**b**) empirical density, (**c**) two dominants Gaussian components, (**d**) JSD between empirical density and mixed density, (**e**) two dominant and K−2 subdominant Gaussian components, (**f**) proposed cluster algorithm, (**g**) marginal density for lung and chest, and (**h**) final mixed density for all components. Note that JSD stands for Jensen–Shannon divergence.

**Figure 6:**
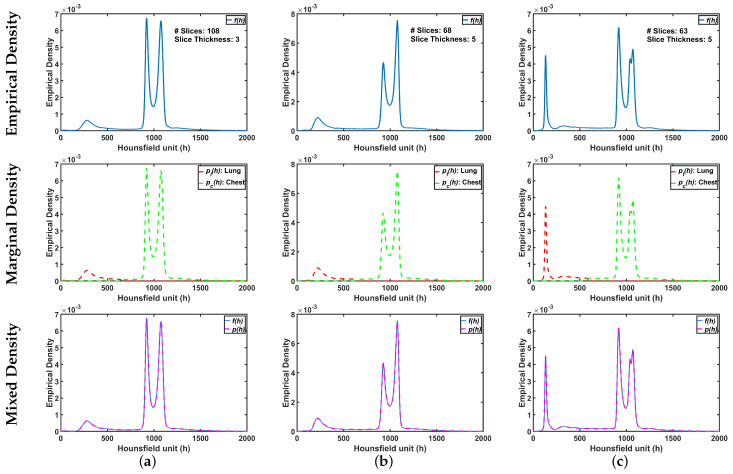
An illustrative example of the proposed appearance model estimated from Thick-Section CT appearance model for (**a**) healthy/mild, (**b**) moderate, and (**c**) severe COVID-19 infected lung. Note that first, second, and third rows represent empirical, marginal, and mixed densities, respectively.

**Figure 7:**
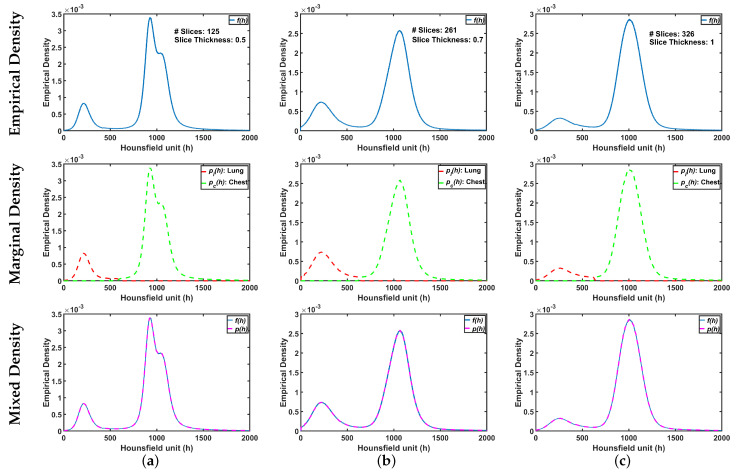
An illustrative example of the proposed appearance model estimated from Thin-Section CT appearance model for (**a**) healthy/mild, (**b**) moderate, and (**c**) severe COVID-19 infected lung. Note that first, second, and third rows represent empirical, marginal, and mixed densities, respectively.

To demonstrate step by step how our proposed approach works, Figure 5b shows empirical density for the 3D CT chest volume (Figure 5a), and the two Gaussian mixtures approximating its dominant modes are presented in Figure 5c. Furthermore, Figure 5c demonstrates the JSD between the empirical density and the two estimated dominant Gaussian components. Figure 5d shows the changes of JSD and the best-estimated number of Gaussian components that are demonstrated in Figure 5e. Figure 5f demonstrated the classification of the subdominant Gaussian components based on the use of the proposed clustering algorithm (Algorithm 2). Figure 5g,h demonstrates the final marginal densities for lung and chest as well as the final estimated mixed density. Figure 6 and Figure 7 demonstrate the ability of the proposed probabilistic model to handle the variability in the empirical density that it may occur due to the severity of infections or the variability of the scanning protocol.

**Figure 8:**
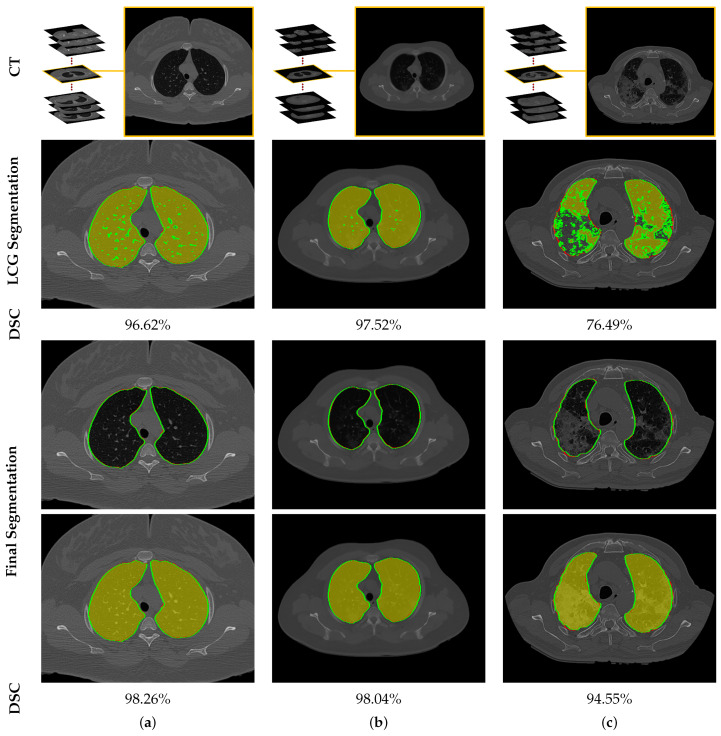
An illustrative example of the proposed segmentation for (**a**) healthy/mild, (**b**) moderate, and (**c**) severe COVID-19 infected lung. Note that red border (green border or yellow region) refers to ground-truth (segmentation).

**Figure 9:**
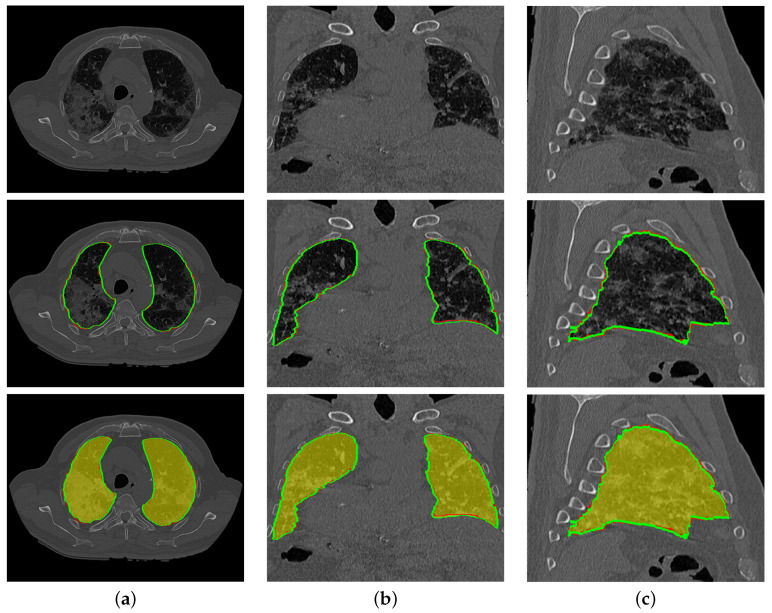
An illustrative example of the proposed segmentation (second and third rows) for a severe COVID-19 infected lung at (**a**) 2D axial, (**b**) coronal, and (**c**) sagittal cross sections of an original image (first row). Note that red border (green border or yellow region) refers to ground-truth (segmentation).

**Figure 10:**
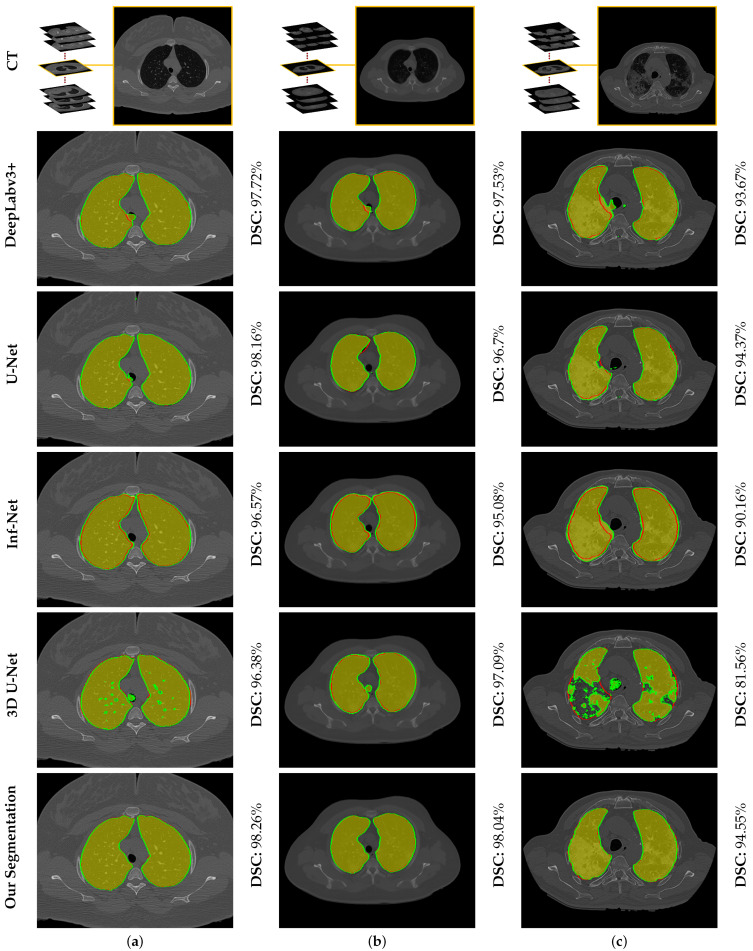
An illustrative example of the proposed segmentation compared to other deep learning approaches for (**a**) healthy/mild, (**b**) moderate, and (**c**) severe COVID-19 infected lung. Note that red border (green border or yellow region) refers to ground-truth (segmentation).

To highlight the promise of including the rotation invariant MGRF-based morphological/anatomical constraints with the adaptive first-order appearance model, the system’s performance is evaluated before and after inclusion, as demonstrated in Table 2. As shown in the table, the proposed segmentation is enhanced after including MGRF-based morphological/anatomical constraints, particularly in severe cases where the DSC is significantly increased from $82.15_{\pm 13.13}\%$ to $95.15_{\pm 1.91}\%$. To more prove the attainable enhancement of the system, Figure 8 presents three examples of the proposed system before and after the inclusion for healthy/mild, moderate, and severe COVID-19 infected lung. As shown in the figure, the developed system outperforms the proposed appearance model alone (i.e., LCG) for three examples, whereas the proposed system shows its ability to segment a severe COVID-19 infection with 94.55% DSC compared to the proposed appearance model which gives 76.49% DSC. Moreover, Figure 9 presents the proposed segmentation for a severe lung COVID-19 infection at different cross-sections (i.e., 2D axial, coronal, and saggital) to visually show the efficiency of the proposed system. Overall, the proposed system achieves a DSC, overlap, BHD, and ALVD of $95.76_{\pm 1.83}\%$, $91.76_{\pm 3.29}\%$, $4.06_{\pm 5.01}$, and $2.93_{\pm 2.39}$, respectively. Finally, to prove the robustness of the proposed segmentation approach, deep learning approaches are adopted as a comparison: DeepLabv3+ [52] using ResNet-50 network as a backbone, Inf-Net [28] with backbone ResNet-50 network, U-Net [25], and 3D U-Net [53]. The results are reported in Table 3. As demonstrated in the table, the 3D U-Net approach gives a worst performance of $66.08_{\pm 35.99}\%$ DSC, $58.30_{\pm 34.81}\%$ overlap, $44.44_{\pm 48.60}$ BHD, and $66.77_{\pm 133.01}$ ALVD, while the proposed segmentation approach gives the best performance compared to these deep learning approaches. Moreover, to visually demonstrate the capability of the proposed system, three different examples of healthy/mild, moderate, and severe lung COVID-19 infections are segmented using these approaches, as presented in Figure 10. As demonstrated in the figure, the proposed approach segments the three examples better than the other four approaches. Moreover, the U-Net approach has a DSC close to the proposed approach. However, there are some parts that segment incorrectly as demonstrated in the figures, e.g., classifying part of trachea or chest as lung. Therefore, the proposed system is much better due to its segmentation being closer to the ground-truth. Therefore, it is highly recommended to use our approach to segment the lung infected by COVID-19 as it shows better performance than the state-of-the-art deep learning approaches. In addition, it is unsupervised technique, thus it will not suffer from the underfitting and overfitting problems.

## 5. Discussion and Conclusions

Experiments demonstrate that the proposed framework is promising and achieved high accuracy, with identification of the first-order appearance model followed by 3D morphological constraints based on analytical estimation MGRF parameters producing good results when segmenting the COVID-19 infected lung region from CT images. Quantitative metrics of accuracy including the DSC, overlap coefficient, 95th-percentile BHD, and the ALVD metrics all show consistent performance on our sample data set of 32 subjects, outperforming current, state-of-the-art deep learning-based lung segmentation approaches. The results herein demonstrate the ability of the developed system to segment lung on a CT image, whose DSC is improved from $89.59_{\pm 9.76}\%$ to $95.67_{\pm 1.83}\%$ when 3D morphological MGRF-based constraints are included in the system pipeline. However, the accuracy of the proposed segmentation system will get affected if the lung is significantly damaged or filled with water, or the appearance of the lung is closed to the chest. Thus, separation based on appearance model will be very challenging task. Therefore, we plan to add some shape model approach in our system to overcome these problems. Moreover, a future extension of this work would integrate the proposed segmentation approach into a computer-aided diagnostic system to assess pulmonary function and risk of mortality in COVID-19 patients, which is the ultimate goal of our research group. Furthermore, the morphological constraints could be made to support large-scale inhomogeneity of the kind seen in severe lung infection. This will be accomplished by expanding the neighborhood system to include larger cliques so that the MGRF model incorporates higher order interaction effects.

## Figures and Tables

**Table 1:** Dataset characteristics.

	Class	Resolution	#Slices	#Patients	Total
*Training*	Healthy/Mild	512×512	43–54	2	34
	Moderate		35–397	20	
	Severe		46–321	12	
*Testing*	Healthy/Mild		51–125	4	32
	Moderate		36–607	17	
	Severe		44–577	11	

**Table 2:** Quantitative evaluation of the proposed segmentation system before and after applying rotation invariant Markov–Gibbs random field (MGRF). Note that LCG, DSC, BHD, and ALVD stand for linear combination of Gaussian, Dice similarity coefficient, 95th-percentile bidirectional Hausdorff distance, and absolute lung volume difference, respectively.

		DSC	Overlap	BHD	ALVD
*LCG-model*	*Healthy/Mild*	$96.37_{\pm 0.47}\%$	$92.99_{\pm 0.88}\%$	$11.32_{\pm 4.59}$	$3.58_{\pm 2.06}$
	*Moderate*	$92.57_{\pm 4.64}\%$	$86.47_{\pm 7.61}\%$	$9.59_{\pm 5.30}$	$7.31_{\pm 7.39}$
	*Severe*	$82.51_{\pm 13.13}\%$	$71.98_{\pm 17.26}\%$	$13.77_{\pm 7.20}$	$23.16_{\pm 19.29}$
	*Overall*	$89.59_{\pm 9.76}\%$	$82.31_{\pm 13.72}\%$	$11.24_{\pm 6.08}$	$12.29_{\pm 14.63}$
*Final System*	*Healthy/Mild*	$97.53_{\pm 0.56}\%$	$95.18_{\pm 1.06}\%$	$2.41_{\pm 1.12}$	$1.72_{\pm 1.09}$
	*Moderate*	$95.54_{\pm 1.91}\%$	$91.53_{\pm 3.41}\%$	$4.25_{\pm 3.73}$	$3.48_{\pm 2.59}$
	*Severe*	$95.19_{\pm 1.66}\%$	$90.87_{\pm 3.01}\%$	$6.70_{\pm 6.97}$	$2.52_{\pm 2.32}$
	*Overall*	$95.76_{\pm 1.83}\%$	$91.76_{\pm 3.29}\%$	$4.86_{\pm 5.01}$	$2.93_{\pm 2.39}$

**Table 3:** Quantitative evaluation of the proposed segmentation system compared with other deep learning approaches. Note that DSC, BHD, and ALVD stand for Dice similarity coefficient, 95th-percentile bidirectional Hausdorff distance, and absolute lung volume difference, respectively.

		DSC	Overlap	BHD	ALVD
*DeepLabv3+* [52]	*Healthy/Mild*	$80.32_{\pm 32.97}\%$	$74.93_{\pm 37.81}\%$	$23.58_{\pm 40.93}$	$116.36_{\pm 220.88}$
	*Moderate*	$95.15_{\pm 1.52}\%$	$90.79_{\pm 2.75}\%$	$8.85_{\pm 21.37}$	$7.32_{\pm 3.68}$
	*Severe*	$93.80_{\pm 2.47}\%$	$88.41_{\pm 4.29}\%$	$27.01_{\pm 46.72}$	$8.78_{\pm 4.24}$
	*Overall*	$92.88_{\pm 11.49}\%$	$88.07_{\pm 13.22}\%$	$16.79_{\pm 34.17}$	$21.41_{\pm 77.87}$
*U-Net* [25]	*Healthy/Mild*	$93.47_{\pm 7.46}\%$	$88.39_{\pm 12.36}\%$	$25.68_{\pm 15.60}$	$5.46_{\pm 6.00}$
	*Moderate*	$95.09_{\pm 2.02}\%$	$90.71_{\pm 3.62}\%$	$19.04_{\pm 35.58}$	$3.61_{\pm 3.36}$
	*Severe*	$91.68_{\pm 5.07}\%$	$84.99_{\pm 8.30}\%$	$44.81_{\pm 73.04}$	$9.41_{\pm 7.24}$
	*Overall*	$93.77_{\pm 4.30}\%$	$88.56_{\pm 7.16}\%$	$28.49_{\pm 50.08}$	$5.76_{\pm 5.80}$
*Inf-Net* [28]	*Healthy/Mild*	$89.77_{\pm 10.41}\%$	$82.56_{\pm 15.81}\%$	$17.41_{\pm 27.32}$	$14.41_{\pm 9.32}$
	*Moderate*	$92.73_{\pm 1.64}\%$	$86.49_{\pm 2.85}\%$	$13.40_{\pm 25.32}$	$13.43_{\pm 3.29}$
	*Severe*	$90.20_{\pm 4.56}\%$	$82.42_{\pm 7.06}\%$	$36.03_{\pm 38.77}$	$14.91_{\pm 5.65}$
	*Overall*	$91.54_{\pm 4.52}\%$	$84.67_{\pm 6.99}\%$	$21.44_{\pm 31.40}$	$14.00_{\pm 4.96}$
*3D U-Net* [53]	*Healthy/Mild*	$78.11_{\pm 35.98}\%$	$72.80_{\pm 39.39}\%$	$29.41_{\pm 41.91}$	$140.67_{\pm 276.19}$
	*Moderate*	$68.46_{\pm 35.82}\%$	$60.58_{\pm 33.85}\%$	$42.04_{\pm 51.53}$	$33.91_{\pm 35.35}$
	*Severe*	$55.72_{\pm 37.95}\%$	$46.95_{\pm 35.31}\%$	$55.25_{\pm 50.43}$	$93.15_{\pm 158.26}$
	*Overall*	$66.08_{\pm 35.99}\%$	$58.30_{\pm 34.81}\%$	$44.44_{\pm 48.60}$	$66.77_{\pm 133.01}$
*Our System*	*Healthy/Mild*	$97.53_{\pm 0.56}\%$	$95.18_{\pm 1.06}\%$	$2.41_{\pm 1.12}$	$1.72_{\pm 1.09}$
	*Moderate*	$95.54_{\pm 1.91}\%$	$91.53_{\pm 3.41}\%$	$4.25_{\pm 3.73}$	$3.48_{\pm 2.59}$
	*Severe*	$95.19_{\pm 1.66}\%$	$90.87_{\pm 3.01}\%$	$6.70_{\pm 6.97}$	$2.52_{\pm 2.32}$
	*Overall*	$95.67_{\pm 1.83}\%$	$91.76_{\pm 3.29}\%$	$4.86_{\pm 5.01}$	$2.93_{\pm 2.39}$

## Data Availability

The data presented in this study are openly available in The Cancer Imaging Archive (TCIA) at https://doi.org/10.7937/TCIA.2020.GQRY-NC81 accessed date 5 June 2021, reference number [50].
